# Spontaneous Splenic Rupture Complicating Severe *P. falciparum* Infection: A Case Report and Literature Review

**DOI:** 10.1155/2019/2781647

**Published:** 2019-10-24

**Authors:** Eltaib Saad, Elamin Elsamani, Walid Abdelrahman

**Affiliations:** ^1^Department of Surgery, University of Khartoum, Khartoum, Sudan; ^2^Mayo University Hospital, Co. Mayo, Castlebar, Ireland; ^3^Sudan Medical Specialization Board, Khartoum, Sudan

## Abstract

Spontaneous rupture of the spleen in malarial infection or malarial splenic rupture (MSR) is a rare but life-threatening condition complicating severe malarial infection in tropics and subtropics, and hence it deserves special attention. A high index of clinical suspicion is warranted for the early diagnosis as delayed or missed diagnosis can be potentially fatal. We report on a 32-year-old male who was diagnosed with severe *Plasmodium falciparum* infection and presented with an acute abdomen due to spontaneous splenic rupture. He was managed conservatively and had a successful outcome. The epidemiology, pathophysiology, clinical presentation, and management of MSR were briefly discussed in this report.

## 1. Introduction

Malaria is a parasitic life-threatening endemic disease in over 100 tropics and subtropics [1]. In 2018, the World Health Organization (WHO) reported about 219 million cases of malaria worldwide [1]. In Sudan, the incidence of malaria was reported to be 9 million cases in the year 2007 with nearly 44,000 malaria-related deaths [2]. The range of clinical presentation of malaria varies widely across various transmission zones from asymptomatic parasitemia and febrile illness to severe life-threatening disease [3].

The role of the spleen in the immune response against malaria is well-studied, especially in endemic regions [4]. Splenomegaly is a common physical finding in malarial infection, and tropical splenomegaly syndrome is a differential diagnosis of splenomegaly in tropics and subtropics [4]. Malarial splenic rupture (MSR) is a rare but potentially fatal complication of severe malarial infection with an estimated incidence of 2% in acute malarial infections [4]. Yet, the exact incidence is largely unknown due to the substantial under-reporting [4–6].

## 2. Case Presentation

A 32-year-old male presented to our emergency department with a 7-day history of dull, aching left-sided upper abdominal pain which was associated with intermittent fever and rigors, night sweating, anorexia, and generalized ache. No history of cough, dyspnea, or chest pain was elicited. He had no diarrhea, jaundice, itching, or epistaxis. He denied any history of trauma. The rest of systemic review was unremarkable. He was a resident from a malaria-endemic region, and he had prior uncomplicated acute malarial infections with *P. falciparum* that were treated with oral antimalarial therapy with unremarkable recovery.

**Figure 1:**
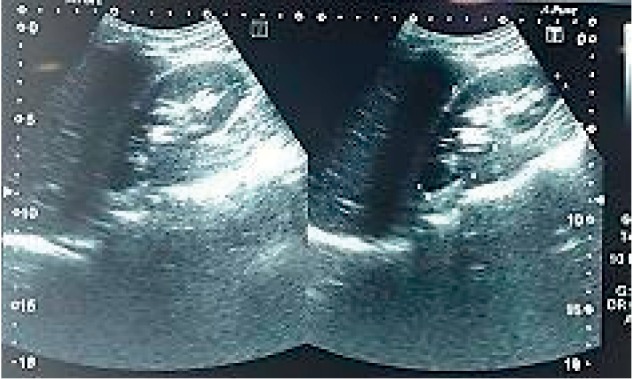
Abdominal ultrasonography showing splenic subcapsular haematoma.

**Figure 2:**
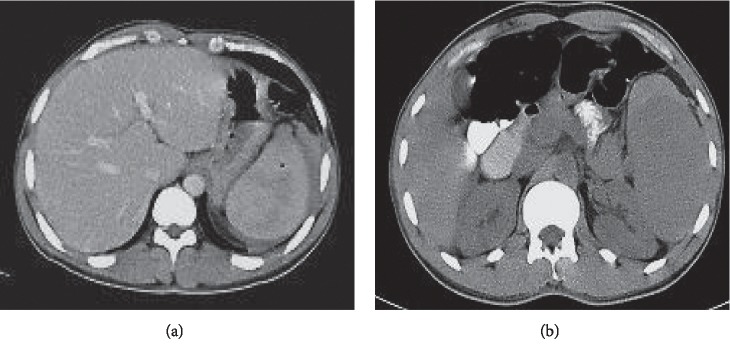
(a, b) Contrast-enhanced abdominal CT demonstrating subcapsular haematoma with grade II splenic rupture.

On physical examination, he looked confused. He was pale but not cyanosed or jaundiced. He was febrile to 39.8°C, tachycardic with a pulse rate of 110 beats per minute in sinus rhythm, and hypotensive to 90/55 mmHg and saturating 91% on room air breathing. There were bilaterally equal breathing sounds on the chest examination. Abdominal examination revealed left upper quadrant (LUQ) tenderness with localized LUQ guarding without signs of generalized peritonitis. He received high-flow oxygen, initial intravenous (IV) fluid resuscitation, and analgesia. Blood cultures were taken, and an empirical dose of broad-spectrum antibiotics was administered as per local sepsis guidelines. Full blood count revealed low haemoglobin of 8 g/dl, white cell count of 5,000/mm^3^, and platelet count of 170,000/mm^3^. The coagulation profile was normal. Serum electrolytes, renal profile, and liver profile were all within normal ranges. Urinalysis and chest X-rays were normal. Peripheral blood smears for malaria demonstrated multiple ring stages of *P. falciparum* with a high parasitemia index (PI) (>5%). He was diagnosed with severe malarial infection evident by circulatory collapse as per WHO guidelines [7], and he was commenced on intravenous quinine (a slow infusion of 600 mg every 8 hours) as per national malaria treatment protocol. Two units of packed red blood cells (RBCs) were transfused. Abdominal ultrasound (US) scan revealed free fluids collection in the pelvis measuring about 9.6 × 7.9 cm and a perisplenic haematoma (Figure 1). No focal splenic lesions were visualized. A contrast-enhanced computed tomography (CT) scan of the abdomen and pelvis demonstrated a 3 cm subcapsular splenic haematoma with free peritoneal fluids indicating grade II splenic rupture (Figures 2(a) and 2(b)). No active bleeding or contrast extravasation was visualized. The liver appearance was remarkably normal. There was no ascites, and the splenic, mesenteric, and portal veins were patent. Severe *P. falciparum* infection was presumed as the underlying cause of spontaneous rupture of the spleen, as there was no evidence of other documented etiologies (i.e., traumatic, haematological, or infectious) for the splenic rupture.

The patient was admitted to a high-dependency unit (HDU) for close observation after stabilization. An attempt to preserve the spleen was planned by the surgical team. The patient was informed about the findings of the scan. He was carefully counselled about a substantial need for an exploratory laparotomy and splenectomy, as a life-saving surgery, if his clinical condition further deteriorated at any time. The consequences of splenectomy, if is deemed necessary according to the consensus of the treating team, were also explained. He agreed to continue with the conservative management. He was transfused with two more units of RBCs. The haemodynamic parameters (pulse rate, blood pressure, urinary output, and central venous pressure measurements) and serial haemoglobin levels remained consistently stable for five consecutive days with IV fluids and blood transfusions only. He was transferred to the general ward on day 5. The patient made a good recovery as fever subsided, and his pain was controlled. A repeat blood film smear on day 7 confirmed parasitic clearance, and quinine therapy was discontinued. He was prescribed a 5-day course of doxycycline (100 mg once daily). A follow-up CT scan on day 10 revealed resolution of the subcapsular splenic haematoma with no residual collections. He was discharged on day 11. He was reviewed after three weeks in the outpatient clinic and reported feeling well with no recurrence of the symptoms. He was discharged from the surgical services and was advised to seek urgent medical advice if similar complaints recurred in the future.

## 3. Discussion

A total number of 252 cases of MSR were reported in the medical literature according to a review conducted by Osman et al. [6]. *P. falciparum* and *P. vivax* species are reported in the vast majority of cases [4–6, 8]. Our reported patient was a 32-year-old male, which is keeping with the literature that reported a median presentation age of 31 years with a male to female ratio of 3 : 1 [6]. He was also a resident from an endemic region, which is in line with the literature that documented the occurrence of MSR even among those living in endemics [6, 9]. Nevertheless, the highest relative incidence of MSR is observed among travelers to the endemics and nonimmunized populations [6]. Of note, MSR was reported in patients who received initial antimalarial therapy and prophylaxis [9, 10].

Splenic reactions to malarial infection encompass a spectrum of pathological findings ranging from reactive splenomegaly to haematoma formation, splenic infarctions, and, rarely, abscess formation [11]. A combination of three principal factors, which ultimately result in subcapsular haematoma formation, is thought to be implicated in pathogenesis of MSR [12]. These three pathological factors are (1) cellular hyperplasia and venous-sinusoidal congestion leading to increased tension and stress on the capsule, (2) vascular occlusion of the reticuloendothelial cells resulting in thrombotic or ischemic events, and (3) episodic increase in intra-abdominal pressure accompanying coughing, sneezing, and laughing which add more stress on the diseased and friable spleen [12]. A minor insult that could be simple physiological activities (like bending for instance) is hypothesized to trigger a capsular tear that eventually evolves to the splenic rupture [5].

In fact, MSR is usually more common in acute primary infections [6] because in chronic or recurrent infections the splenic enlargement is more gradual, and subsequently, the tension within the capsule would fairly be less pronounced [12]. Additionally, the fibrous tissue, resulting from healing of previous attacks, tends to make splenic rupture in recurrent cases less likely [12]. We do not have an explanation for our patient's presentation with a splenic rupture despite having prior documented episodes of uncomplicated malarial infections with the same causative species (*P. falciparum*).

The time interval from the onset of fever to splenic rupture ranges widely from 0 to 37 days, with a median of 5 days [6]. The clinical features of MSR include systemic signs which are related to circulatory compromise due to intra-abdominal bleeding and loss of intravascular volume and local signs resulting from localized peritonitis as observed in our patient [4, 6]. Nevertheless, the local signs can be absent in up to the half of cases, and the diagnosis can subsequently be delayed or even missed [6]. Therefore, a high index of clinical suspicion is warranted in patients with severe malaria who develop refractory hypotension to outrule MSR [11]. The CT scan is the gold standard for the confirmation of diagnosis with a sensitivity and specificity of 95% [4].

Splenectomy was historically performed for all MSR cases. In the modern surgical practice era, a nonoperative management can be safely followed for haemodynamically stable patients and splenectomy is indicated for haemodynamically unstable patients with ongoing shock as a critical life-saving surgery [6]. The conservative management includes maintenance of haemodynamics and haemoglobin level by IV fluids and transfusion of blood and blood products, pain control, and a strict bed rest (from 7 to 19 days) [6]. Patients who failed to maintain haemodynamics and haemoglobin level despite appropriate resuscitation measures should be considered for partial or total splenectomy [4, 6]. At present, the documented success rate of the conservative management of MSR is 71% [6]. An algorithm for management of MSR was formulated by Osman et al. [6]. Ultrasonography or CT imaging is used to assess the healing of the ruptured spleen which is usually resolved within two to three weeks [4]. Angiography with embolization of the splenic artery or its branches, while rarely available, can be attempted in haemodynamically stable patients with radiological evidence of active bleeding [13]. Safe and well-resourced settings that allow a meticulous observation and a timely surgical intervention are mandatory if the conservative approach is planned to be followed [6].

The concept of splenic preservation—in haemodynamically stable patients with no evidence of active bleeding—has been recently advocated by many authors to avoid the potential complications of splenectomy [5, 6]. It is important to emphasize on the immune functions of the spleen that include, for instance, immunoprotection against certain serious parasitic and bacterial infections [4–6, 11]. Therefore, the benefits and potential risks of splenectomy have to be carefully assessed by the treating surgeons, and likewise discussed with the patients as part of patient-centered care.

## 4. Conclusion

MSR is a rare but serious surgical complication of severe malarial infection. A high index of clinical suspicion is warranted, especially in endemic regions, as delayed or missed diagnosis can potentially be fatal. A conservative management of MSR for haemodynamically stable patients can be successful as demonstrated in the case.
